# Maturity onset diabetes of the young due to *HNF1A* variants in Croatia

**DOI:** 10.11613/BM.2018.020703

**Published:** 2018-04-15

**Authors:** Tamara Pavić, Agata Juszczak, Edita Pape Medvidović, Carla Burrows, Mario Šekerija, Amanda J Bennett, Jadranka Ćuća Knežević, Anna L Gloyn, Gordan Lauc, Mark I McCarthy, Olga Gornik, Katharine R Owen

**Affiliations:** 1 Department of Biochemistry and Molecular Biology, Faculty of Pharmacy and Biochemistry, University of Zagreb, Zagreb, Croatia; 2 Oxford Centre for Diabetes, Endocrinology and Metabolism, Churchill Hospital, University of Oxford, Oxford, UK; 3 Oxford NIHR Biomedical Research Centre, University of Oxford, Oxford, UK; 4 Vuk Vrhovac University Clinic, Merkur University Hospital, Zagreb, Croatia; 5 Croatian Institute of Public Health, Zagreb, Croatia; 6 School of Medicine, Andrija Štampar School of Public Health, University of Zagreb, Zagreb, Croatia; 7 Department of Clinical Chemistry and Laboratory Medicine, Merkur University Hospital, Zagreb, Croatia; 8 Wellcome Trust Centre for Human Genetics, Oxford, UK; 9 Genos Ltd, Glycobiology Division, Zagreb, Croatia

**Keywords:** *HNF1A*, maturity onset diabetes of the young (MODY), monogenic diabetes, prevalence study

## Abstract

**Introduction:**

Maturity onset diabetes of the young due to *HNF1A* mutations (HNF1A-MODY) is the most frequent form of monogenic diabetes in adults. It is often misdiagnosed as type 1 or type 2 diabetes, but establishing genetic diagnosis is important, as treatment differs from the common types of diabetes. HNF1A-MODY has not been investigated in Croatia before due to limited access to genetic testing. In this study we aimed to describe the characteristics of young adults diagnosed with diabetes before the age of 45 years, who have rare *HNF1A* allele variants, and estimate the prevalence of HNF1A-MODY in Croatia.

**Materials and methods:**

We recruited 477 C-peptide positive and beta cell antibody negative subjects through the Croatian Diabetes Registry. *HNF1A* was sequenced for all participants and systematic assessment of the variants found was performed. The prevalence of HNF1A-MODY was calculated in the study group and results extrapolated to estimate the proportion of diabetic individuals with HNF1A-MODY in Croatia and the population prevalence.

**Results:**

Our study identified 13 individuals harbouring rare *HNF1A* allelic variants. After systematic assessment, 8 were assigned a diagnosis of HNF1A-MODY. Two individuals were able to discontinue insulin treatment following the diagnosis. We estimated that HNF1A-MODY in Croatia has a prevalence of 66 (95% CI 61 - 72) cases *per* million.

**Conclusions:**

The estimated prevalence of HNF1A-MODY in Croatia is similar to that reported in other European countries. Finding cases lead to important treatment changes for patients. This strongly supports the introduction of diagnostic genetic testing for monogenic diabetes in Croatia.

## Introduction

Maturity-onset diabetes of the young (MODY) is a clinically heterogeneous group of disorders, caused by mutations in one of 13 different genes (*1*). One of the most frequent forms of MODY is associated with variants in the *HNF1A* (hepatocyte nuclear factor 1-alpha) gene on chromosome 12, also known as HNF1A-MODY. *HNF1A* is expressed in beta cells, where it encodes a crucial member of the auto-regulatory transcriptional network during embryonic development of pancreas (*2*). In adult life, protein HNF1A regulates the expression of numerous beta cell genes, such as insulin, pyruvate kinase and the glucose transporter molecule *Glut2* (*3*).

Subjects harbouring disease-causing *HNF1A* allelic variants typically present with prominent family history of diabetes (autosomal dominant mode of inheritance), early onset of diabetes (classically before the age of 25 years) and insulin independence evidenced by presence of C-peptide, often despite a long duration of diabetes (*4*). The majority of subjects with MODY do not show signs of insulin resistance and their body mass index (BMI) is in the normal range. The disease is characterized by progressive deterioration of beta cell function, due to an inability to increase insulin secretion in response to hyperglycaemia (*5*). Disease-causing variant alleles in *HNF1A* demonstrate a high penetrance, so 63% of variant carriers develop diabetes before 25 years of age, and 96% before the age of 55 years (*6*).

MODY is frequently misdiagnosed as type 1 or type 2 diabetes due to low awareness among primary care physicians, lack of widely accepted diagnostic protocols and/or unavailability of genetic testing. Definitive diagnosis of HNF1A-MODY is established by sequencing of *HNF1A*, although confirmation that allelic variants are disease-causing remains a challenge. A correct molecular diagnosis is of utmost importance, since subjects with HNF1A-MODY are usually very sensitive to oral treatment with sulphonylurea derivatives (SU) such as gliclazide, which can provide excellent diabetes control for decades (*7*). Glinides have similar mechanism of action to SU, therefore are an alternative treatment for HNF1A-MODY (*8*). Establishing a genetic diagnosis of HNF1A-MODY may lead to treatment changes for previously misdiagnosed patients, even to the discontinuation of assumed life-long insulin treatment. Correctly identifying MODY subjects also allows the recognition of affected family members, thereby enabling better disease management and clinical care for these individuals too.

The current diagnostic pathways for MODY, which mostly include clinical criteria, miss many cases through low sensitivity (*9*, *10*). Maturity-onset diabetes of the young is rarely diagnosed in Croatia because genetic testing is not available to most clinicians. Therefore, the prevalence of MODY in Croatia has never been studied. The goal of the study was to describe the clinical characteristics of Croatian subjects with HNF1A-MODY, estimate the prevalence of HNF1A-MODY within Croatian subjects diagnosed with diabetes as young adults, increase the awareness of this specific type of diabetes and thus provide support for introduction of genetic testing for monogenic diabetes in Croatia.

## Materials and methods

### Subjects

Initial recruitment was performed using data from the Croatian national registry of patients with diabetes (CroDiab); a registry established to improve health care, to determine the prevalence and incidence of diabetes and its complications, as well as to monitor morbidity and mortality at the national level (*11*). All primary and secondary care physicians who provide diabetes care were obliged to supply data on their patients to the Vuk Vrhovac University Clinic for Diabetes, Endocrinology and Metabolic Diseases on an annual basis until 2015 (*12*). Data supplied constitutes a basic informatic sheet, recognised by the international diabetology community as optimal data collection form for the follow-up and improvement of diabetes care.

All adult subjects with diabetes onset under age of 45 years and currently older than 18 years were eligible to take part in the study. Other inclusion criteria encompassed the following: evidence of endogenous insulin secretion (fasting or random C-peptide ≥ 0.2 nmol/L) and negative glutamic acid decarboxylase antibodies (GADA) and islet cell autoantibodies (ICA). Letters of invitation, describing the protocol and aims of the study, were sent to the home address of eligible subjects. Subjects newly diagnosed with diabetes who met inclusion criteria were also invited to participate. Study participants were recruited at Vuk Vrhovac University Clinic for Diabetes, Endocrinology and Metabolic Diseases, Merkur University Hospital, Zagreb; the Reference Centre for Diabetes of the Croatian Ministry of Health, in the period between June 2013 and June 2015. Whole blood and plasma samples were collected from every patient. Blood was collected into EDTA-treated tubes and plasma was separated by centrifugation. All samples were stored at -20 °C until further analysis.

Data collection included the following information: age, sex, age of diabetes onset, treatment history, current medication, family history of diabetes, anthropometrical data (weight, height, body mass index (BMI), blood pressure) and biochemical data (fasting glucose, glycated haemoglobin (HbA_1c_), C-peptide, lipid profile, creatinine). All recruited participants gave written informed consent. The study was approved by Ethics Committee of Merkur University Hospital and Faculty of Pharmacy and Biochemistry, University of Zagreb, Croatia. The study was conducted in accordance with the Declaration of Helsinki.

### Study design

**Figure 1:**
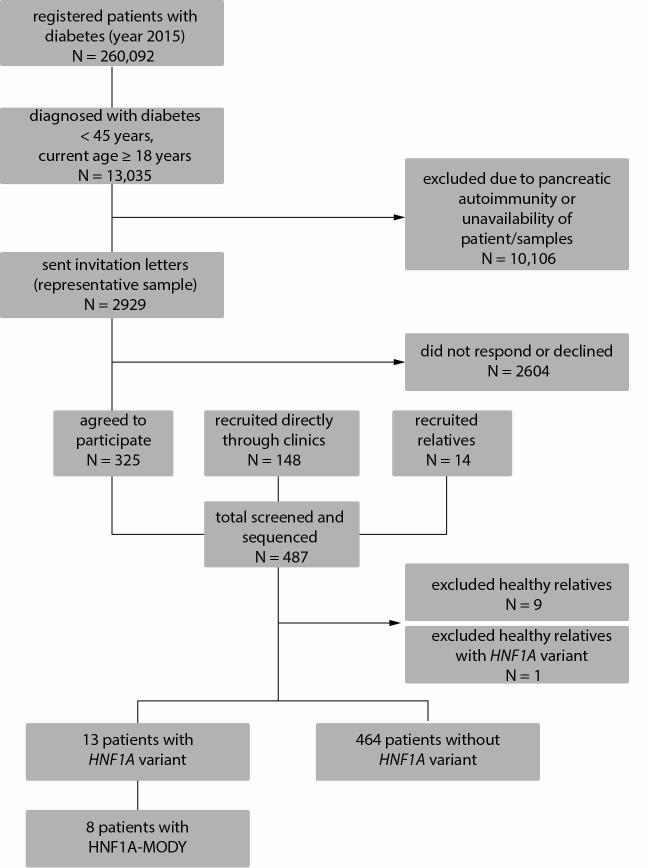
Flowchart showing the study recruitment strategy

Figure 1 illustrates the study recruitment strategy. Study participants were mainly recruited through CroDiab registry. According to the study inclusion criteria there were 13,035 adult subjects with diabetes onset before the age of 45 years. Patients in CroDiab were prioritized for invitation if GADA was analysed at the Reference Centre for Diabetes, which participated in an antibody standardization programme. From the list of eligible individuals, 2929 subjects who fulfilled the inclusion criteria were invited to participate from throughout Croatia. For subjects without existing beta cell antibody analysis, the antibodies were assayed prior to the inclusion into the study. Three hundred and twenty-five subjects were recruited; others did not respond or declined to participate. A further 148 recently diagnosed subjects were recruited during their visit to the Vuk Vrhovac University Clinic. Later on, additional 14 family members were recruited for co-segregation studies. Of these, 10 did not have diabetes, resulting in a total of 477 individuals with diabetes recruited in the study.

## Methods

### Biochemical and immunological assays

Plasma glucose and serum lipid profile were assayed using routine enzymatic methods (AU680, Beckman Coulter, Brea, USA) in the accredited (ISO15 189:2012) laboratory. HbA_1c_ was measured by turbidimetric immuno-inhibition traceable to the IFCC-reference system and reported in DCCT-aligned units (AU680, Beckman Coulter, Brea, USA), with a total imprecision (CV) of 1.8%. C-peptide was analysed using a chemiluminescence immunoassay on the Advia Centaur XP analyser (Siemens Healthcare Diagnostics, Eschborn, Germany) with a CV of 5.6%. Islet cell antibodies were measured by indirect immunofluorescence. Rate of positivity was calculated by determining end-point titres of samples that were converted to the units of Juvenile Diabetes Foundation (JDF-U) by comparison with a standard curve of log 2 JDF units with log 2 of end-point titre of standard sera. The threshold of detection was > 5 JDF units. Glutamic acid decarboxylase antibodies were measured by enzyme-linked immunosorbent assay (ELISA) (Euroimmun AG, Lübeck, Germany). The cut-off for the positive result was set to 5 WHO Units/mL. Analysis were performed in a laboratory participating in the international harmonisation programme (DASP; Diabetes Antibody Standardization Program) (*13*).

#### HNF1A sequencing and analysis of the HNF1A variants

Deoxyribonucleic acid (DNA) was extracted from the whole blood sample using QIAmp DNA Mini Blood kit (Qiagen, Hilden, Germany), according to the manufacturer’s protocol (*14*).

Quantity and quality of the extracted DNA were evaluated using Nanodrop 8000 UV-Vis spectrophotometer (Thermo Scientific, Waltham, USA). Both were satisfactory, with an average DNA concentration of 38.69 (6.49 – 107.60) ng/µL and average A260/A280 ratio of 1.84 (1.46 – 2.66).

Extracted genomic DNA (2.5 µg) was amplified by polymerase chain reaction and sequenced bidirectionally by Sanger sequencing, using forward and reverse primers for all 10 *HNF1A* exons. Primers were ordered from Eurofins MWG Operon (Ebersberg, Germany) according to a published sequence (*15*). Sequencing was performed at the Diabetes Research Laboratory of the Oxford Centre for Diabetes, Endocrinology and Metabolism, UK. The final sequencing read was obtained using ABI 3730 capillary sequencer (Aplied Biosystems, Birchwood, UK). The frequency of known common variants such as p.L17L, p.I27L, and p.A98V was compared to their minor allele frequency (MAF) in the 1000 Genomes database (0.44 *vs.* 0.41, 0.29 *vs.* 0.30, 0.05 *vs.* 0.02, respectively). Variants p.I27L and p.A98V were also genotyped and had a mean of 1.1% and 0.3% discordance rate, when compared to the Sanger sequencing result.

**Figure 2:**
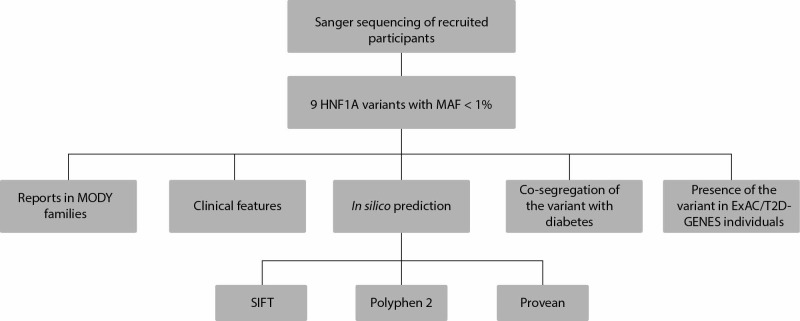
Systematic assessment of identified *HNF1A* variants. MAF - minor allele frequency. MODY - Maturity-onset diabetes of the young. SIFT - Sorting intolerant from tolerant.

Analysis of sequencing was performed in Mutation Surveyor version 5.1 (Soft Genetics, Cambridge, UK). Disease causality of variants was assessed taking into account clinical features, co-segregation of diabetes in affected family members (where available) and *in silico* analysis of missense variants using SIFT (Sorting Intolerant From Tolerant), Polyphen2 (PPH2) and Provean bioinformatics tools (Figure 2). Identified variants were also assessed for their presence in publically available databases (Exome Sequencing Aggregation Consortium, ExAC, Broad Institute, Boston, USA) and in the 12,940 exomes from the T2D-GENES study comprising participants with type 2 diabetes mellitus (T2D) and non-diabetic controls which are also included in ExAC populations (http://www.type2diabetesgenetics.org). Although details of diabetic status are not available for ExAC participants and it would not be impossible for a patient with MODY to be included, in general, presence of the variant in a population cohort would suggest that the variant is not disease-causing.

### Statistical analysis

Non-parametric summary measures were used to describe the groups (subjects with a rare *HNF1A* allelic variant (N = 13) and without the *HNF1A* variants (N = 464)). Medians with ranges (min - max) were calculated for non-parametric continuous variables (current age, age at diagnosis, duration of diabetes, BMI, HbA_1c_ and C-peptide, lipid profile) and percentages for categorical measures (gender and current treatment). Non-parametric statistics (Mann-Whitney test) were used for comparison of continuous variables among groups. Differences of frequencies for categorical variables were tested using the Chi-squared (treatment) and Fisher’s test (gender). Significant differences were assumed for P < 0.05. The prevalence of HNF1A-MODY among subjects with diabetes was calculated as the proportion of individuals with HNF1A-MODY out of the total number of participants and extrapolated to calculate estimated population prevalence using the latest available data (year 2015) from the Croatian Institute of Public Health. Statistical analysis was performed using SPSS v23.0 (IBM, Armonk, USA).

## Results

### Characteristics of subjects

Basic anthropometric and clinical data of the recruited subjects are given in Table 1. None of the participants were known to have monogenic diabetes before the study. Subjects with diabetes and a rare *HNF1A* allelic variant were predominantly females (P = 0.02). Their median (min - max) age (including both probands and affected relatives) was 48 (18 - 72) years, their median age at diagnosis of diabetes was 35 (16 - 50) years and their median diabetes duration was 10 (2 - 31) years. We found no significant differences between diabetic subjects with or without a rare *HNF1A* allelic variant regarding their current age, age at onset and duration of diabetes. The subjects with a rare *HNF1A* variant had lower median BMI compared to subjects without a rare *HNF1A* variant (P = 0.003). Lipid profile also showed differences between the groups. Median HDL-cholesterol was higher (1.4 *vs.* 1.2 mmol/L, P = 0.024) and median triglycerides were lower (1.1 *vs.* 1.6 mmol/L, P = 0.015) in those with *HNF1A* variants compared to those without.

**Table 1:** Characteristics of the study participants

	**Diabetes and no rare *HNF1A* variant**	**Diabetes and rare *HNF1A* variant**	**P-value^†^**
N	464	13*	
Gender (males, % or proportion)	58	3/13	0.020
Age (years)	47 (18 - 78)	48 (18 - 72)	0.571
Age at diagnosis (years)	37 (5 - 47)	35 (16 - 50)	0.142
Duration of diabetes (years)	11 (0 - 45)	10 (2 - 31)	0.444
BMI (kg/m^2^)	29.6 (15.5 - 53.2)	24.1 (19.1 - 38.4)	0.003
Current treatment:			0.005
- Oral glucose-lowering agents (% or proportion)	52	2/13	NP
- Insulin therapy (% or proportion)	13	3/13	NP
- Insulin + oral agents (%)	23	2/13	NP
- Diet only (% or proportion)	11	5/13	NP
- Unavailable/without therapy (%)	2	1/13	NP
HbA_1c_ (%)	7.3 (3.9 - 14.2)	6.8 (5.3 - 11.9)	0.094
C-peptide (nmol/L)	0.73 (0.21 - 3.81)	0.33 (0.22 - 1.56)	0.006
Continuous variables are given as median and interquartile range, age is given as median (min - max), while categorical variables are given as percentages. *The number excludes one recruited relative with a rare *HNF1A* allelic variant, but without diabetes.^†^P-values were calculated using Mann-Whitney U test (significance level α = 0.05); for categorical variables Chi-square (treatment) and Fisher’s (gender) tests were used. NP - statistical analysis was not performed due to too small sample size. BMI - body mass index. HbA_1c_ - glycated haemoglobin.			

Current medical treatment differed significantly between the subjects with a rare *HNF1A* variant and subjects without a rare *HNF1A* variant (P = 0.005). The majority of the subjects with a rare *HNF1A* allele variants received dietary intervention only (39% compared to 11% in the group without an *HNF1A* variant), while the majority of subjects without the variants were treated with oral glucose-lowering agents only (51.7% compared to 15.4% in HNF1A group). Among the subjects with a rare *HNF1A* variant, one was treated with a sulphonylurea derivative and two with a glinide.

We observed no significant difference in HbA_1c_ values between the groups, but subjects with diabetes and an *HNF1A* variant had lower C-peptide values (P = 0.006).

Among subjects with an *HNF1A* variant, 12 had at least a two-generational family history of diabetes, whilst detailed family histories were unavailable for one proband due to loss of contact with relatives.

### HNF1A variants identified in the study

Sequencing of *HNF1A* gene identified 9 rare non-synonymous *HNF1A* allelic variants, including 3 protein truncating (PTV) and six missense variants (Table 2), in 9 probands and 5 relatives (1 without diabetes).

**Table 2:** Molecular and clinical characteristics of subjects with rare *HNF1A* variants identified in this study

**Study ID**	**DNA variant**	**Protein variant**	**Variant type**	**Reported in literature**	**Bioinformatics prediction on variant functional effect**			**Age at DM diagno-** **sis**	**Number of genera tions with DM**	**BMI^‡^**	**Treatment^‡^**	**Allele frequency in ExAC**
**SIFT**	**PPH2**	**Provean**
496	c.8C>G	S3C	missense	no	D	D	D	37	1	24.17	insulin + glinide	0
042	c.451G>A	G151S	missense	no	D	D	D	35	2	26.37	metformin+ SU+DPP4I	0
294	c.685C>T	R229X	nonsense	yes (*14*)	protein truncating			25	2	19.05	glinide	0
466*								36	3	N/A	insulin	
245	c.751G>A	A251T	missense	yes (*15*)	N	D	N	39	2	27.14	diet	0
343*								50	2	23.80	diet	
345*								53	2	23.88	IFG, diet	
083	c.862G>T	G288W	missense	no^†^	N	D	N	38	2	38.40	diet	0.0001
337*								no DM	2	30.86	no DM	
379	c.872dup	G292 fsdupC	frameshift	yes (*14*)	protein truncating			23	2	23.95	insulin	0
380	c.1136_ 1137delCT	P379 fsdelCT	frameshift	yes (*14*)	protein truncating			16	un- known	22.28	diet	0
055	c.1136C>G	P379R	missense	yes (*14*)	D	D	D	28	2	19.94	insulin	0
385*								N/A	2	29.69	N/A	
072	c.1544C>T	T515M	missense	no†	D	D	N	33	2	27.99	insulin	0.00002
*Affected relative. ^†^Different amino acid reported in this position in subject with diabetes in the literature. ^‡^At median 10 years from diagnosis of diabetes. DM - diabetes mellitus. BMI - body mass index. IFG - impaired fasting glucose. SIFT - Sorting Intolerant From Tolerant. PPH2 - Polyphen 2. N – predicted as neutral. D - predicted as damaging. SU – sulphonylurea derivatives. DPP4I - dipeptidyl peptidase 4 inhibitor. N/A - not available.												

The PTVs and the protein variant p.P379R were previously reported in subjects with HNF1A-MODY, while the protein variant p.A251T was identified previously in a patient with SU-sensitive diabetes (*16*, *17*). Protein variants p.G288W and p.T515M have not been previously reported in MODY families. However, variants with a different amino acid change at the same position (namely, p.G288C and p.T515K) were previously reported as causing MODY (*16*). Two HNF1A protein variants (p.S3C, p.G151S) were novel.

All three bioinformatics tools (SIFT, PPH2 and Provean) predicted both novel variants and p.P379R to be functionally damaging. Variant p.T515M was predicted as damaging by two bioinformatics tools (SIFT and PPH2), while Provean predicted it as neutral. Variants p.A251T and p.G288W were predicted as neutral by SIFT and Provean; on the other hand PPH2 predicted them as damaging.

Two out of nine identified allelic variants were present in the individuals from ExAC (c.862G>T, p.G288W and c.1544C>T, p.T515M) and none of the allelic variants were present in the non-diabetic individuals from T2D-GENES project.

**Figure 3:**
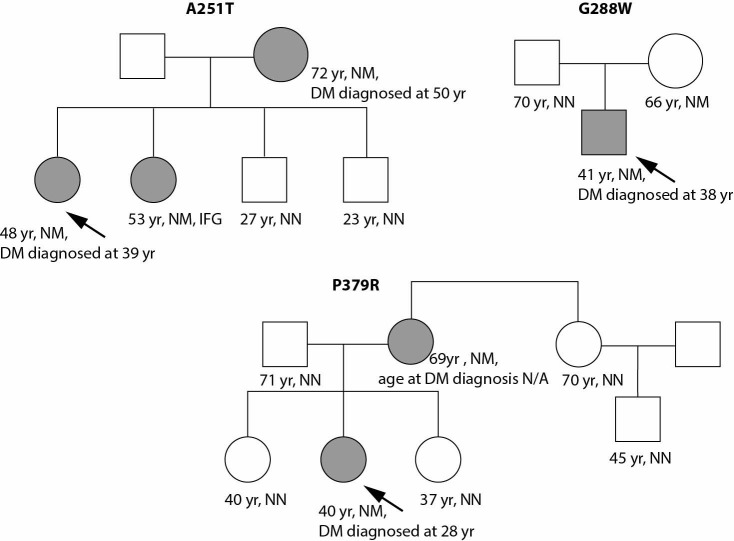
Family trees of probands with variant A251T, G288W and P379R. Probands marked with an arrow; shaded – affected with diabetes; NM - tested and mutation found; NN - tested and no mutation found. IFG - impaired fasting glucose. N/A - not available. yr - years.

Family data was available for 3 families: those with protein variants p.A251T, p.P379R and p.G288W (Figure 3). The p.A251T variant co-segregated with a milder form of dysglycaemia not typical for MODY. The diabetic mother of the proband with p.P379R has the same variant, while the other non-diabetic family members do not carry the variant. The mother of the proband with p.G288W carries the same variant, but does not have diabetes at age of 66 years, despite being obese. Unfortunately, it was not possible to perform co-segregation studies of the novel variants with diabetes due to the unavailability of other family members.

Based on the evidence discussed above, we showed that assigning disease-causality of the identified rare variant is complex and it may not be possible to provide a definitive answer. Taking all above into account, we concluded that six probands have disease-causing or likely disease-causing protein variants (p.S3C, p.G151S, p.R229X, p.G292fs, p.P379fs and p.P379R). The protein variants p.G288W and p.T515M have weak evidence of causing a MODY phenotype. Protein variant p.A251T remains of unknown significance, and may be associated with reduced penetrance of dysglycaemia.

### The prevalence of HNF1A-MODY in Croatia

In this study 13 probands and family members out of 477 individuals with C-peptide positive and beta cell antibody negative diabetes, diagnosed before 45 years of age, had a rare *HNF1A* allelic variant. As discussed above, 8 subjects (6 probands and 2 family members) carried allelic variants that had good evidence for causing MODY. The remaining 5 individuals have variants which may be of lower disease penetrance or increase risk of type 2 diabetes, without causing a MODY phenotype. Therefore, 2.7% (95% CI 1.25 - 4.15%) of our study participants carry rare *HNF1A* allele variants and 1.7% (95% CI 0.54 - 2.86%) have HNF1A-MODY.

The Croatian Institute of Public Health records from 2015 state that in total there were 260,092 individuals registered with diabetes in Croatia, 90% with T2D, and that 7.3% of subjects with T2D had diabetes onset before the age of 45 years. Therefore, this would suggest approximately 281 (95% CI 257 - 304) HNF1A-MODY cases in Croatia and a prevalence of 66 (95% CI 61 - 72) cases per million of population.

### Clinical outcome of the study

Among 4 study participants identified to have HNF1A-MODY and treated with insulin, 2 were changed from insulin to an SU treatment and their diabetes remains well controlled. The remaining 2 had significantly longer durations of diabetes (26 and 30 years), therefore, it was considered by the clinician that a change of therapy would not be appropriate, so they continued receiving insulin treatment.

## Discussion

This is the first study to identify individuals with monogenic diabetes in Croatia. We identified 13 subjects with 9 different rare *HNF1A* allelic variants in 477 individuals with C-peptide positive and beta cell antibody negative diabetes, diagnosed before 45 years of age. Eight of them have HNF1A-MODY. On this basis, we have calculated that 1.7% (95% CI 0.54 - 2.86%) of subjects in our study group have HNF1A-MODY, which corresponds to a prevalence of 66 (95% CI 61 - 72) cases *per* million of population.

In the literature, estimations of the proportion of all MODY cases (including other causative genes) vary between 0.14% and 4.2% of all reported diabetes cases (*9*, *18*–*23*). In 1981, a German study reported a prevalence of clinical MODY of 0.14% of total 40,927 diabetes cases, which corresponded to 70 cases *per* million of the population (*19*). Within the Norwegian HUNT2 study of 1972 subjects with diabetes, a minimum prevalence of HNF1A-MODY of 0.4% was calculated, corresponding to 63 cases *per* million (*21*). UK studies reported a minimum MODY prevalence of 108 *per* million using data from the diagnostic sequencing laboratory (half of the cases had HNF1A-MODY) and 84 cases *per* million from a population-based approach (only HNF1A-MODY) (*9*, *22*). These results from other European countries, calculated with various clinical or sequencing approaches, are comparable to our estimations.

The advantage of our study versus most of the so far published is the fact that all C-peptide positive and beta cell antibody negative participants diagnosed up to age of 45 years had *HNF1A* sequencing, so fewer cases were likely to be missed by narrowing of selection criteria for genetic testing. The “classic” clinical criteria for MODY diagnosis include onset of diabetes before age of 25 years, insulin-independence and at least two-generational family history of diabetes (*24*–*26*). Using this generally accepted cut off for diabetes onset we would miss more than three quarters of the HNF1A-MODY subjects identified in our study.

Despite having no difference in age of onset from the young adults without an *HNF1A* allelic variant, those with rare *HNF1A* allelic variants otherwise had typical features of MODY: they were leaner, with lower C-peptide in keeping with beta cell defect and had less dyslipidaemia.

A limitation of our study is the uncertainty that we selected a representative sample of all young adult diabetes onset individuals in Croatia. Although individuals throughout Croatia were invited, they needed to travel to Zagreb to be recruited, so the participation of non-Zagreb residents was limited. A further limitation of this study is that we did not use a gene-dosage method such as multiplex ligation-dependent probe amplification (MLPA) to detect larger deletions which are missed in Sanger sequencing. However, this is relatively uncommon cause (estimated 3%) of HNF1A-MODY (*27*). Another limitation is difficulty in absolutely assigning disease causality to an identified allelic variant. This is a challenge in many genetic disorders. For instance, allelic variants that were previously perceived as disease-causing have now been identified in populations comprising mostly healthy individuals, which leads us to question their pathogenicity. Careful assessment of identified variants will inform this process. Formal functional assessment of the variants we identified would provide further information about the likely effects, but may not completely define the wide range of phenotypes, which can be associated with the same variant (*28*). We also had difficulty in recruiting family members of the probands to establish co-segregation of the variant with diabetes for all the *HNF1A* variants found. This is likely to reflect the “real world” experience of clinicians, attempting to assess variants found through genetic testing, and reinforces the importance of diagnostic laboratories in collating data on variants of uncertain significance.

A final limitation is that we have investigated the prevalence of HNF1A-MODY only, as the most common form of monogenic diabetes, so the prevalence of other MODY subtypes, such as GCK-, HNF4A- or HNF1B-MODY in Croatia remains unknown.

The exact prevalence of MODY can be determined only by population-based sequencing data, however, this is unlikely to be available in the near future.

In conclusion, in this study we calculated a minimum prevalence of 66 (95% CI 61 - 72) HNF1A-MODY cases per million of population in Croatia, which is consistent with the estimated prevalence of this form of monogenic diabetes in other European countries. Currently, the vast majority of HNF1A-MODY cases in Croatia remain unidentified, therefore the affected individuals are not receiving optimal management. Further advantage of establishing a molecular diagnosis is cascade screening of relatives, with potential change in the treatment of those with diabetes and giving a prognostic advice to currently non-diabetic carriers. In this study two individuals were able to discontinue insulin for oral hypoglycaemia treatment, demonstrating the potential benefits of making a molecular diagnosis. We believe that our study strongly supports the introduction of genetic testing for MODY in Croatia. Further work including health economic evaluation would be required to establish an appropriate pathway.
